# Transcriptomic insights into the immune dynamics of wild-type mice challenged with SARS-CoV-2 Beta variant

**DOI:** 10.1186/s42826-025-00264-4

**Published:** 2026-01-28

**Authors:** Hamid Reza Jahantigh, Amany Elsharkawy, Komal Arora, Chinonye Dim, Mukesh Kumar

**Affiliations:** 1 Department of Biology, College of Arts and Sciences, Georgia State University, Atlanta, GA USA; 2 Center of Diagnostics and Therapeutics, Georgia State University, Atlanta, GA USA

**Keywords:** SARS-CoV-2 Beta variant, B.1.351, RNA-seq, Wild-type mice, Inflammation

## Abstract

**Background:**

Mice are useful small animal models to study the pathogenesis of SARS-CoV-2 infection. As the ancestral SARS-CoV-2 strains did not utilize murine Ace2 as a receptor, wild-type mice were not susceptible to the SARS-CoV-2 infection. Infection of human ACE2-expressing transgenic mice with SARS-CoV-2 induces fatal encephalitis, which is not commonly observed in humans. We and others have previously demonstrated the ability of the SARS-CoV-2 Beta variant to productively infect wild-type mice. Herein, we employed RNA-seq to investigate the transcriptomic landscapes in the lungs after the infection of wild-type mice with SARS-CoV-2 Beta variant.

**Methods:**

We intranasally infected 6-week-old wild-type C57BL/6J mice with the SARS-CoV-2 (B.1.351 strain) and collected lungs at 3- and 6-days post-infection for RNA-sequencing. We used the Limma-Voom package to identify differentially expressed genes (DEGs) and the fgsea package for pathway enrichment analysis. We used Cytoscape to identify hub genes and gene networks. Lastly, we employed RT-qPCR and multiplex assay to validate the RNA-seq data.

**Results:**

Using a cutoff of an adjusted p-value below 0.05 and an absolute log2 fold change value greater than 0.75, we identified 285 DEGs on day 3 and 46 DEGs on day 6. The canonical pathways analysis showed that several key pathways such as apoptosis and cytokine response were upregulated in the infected lungs. Protein-protein interaction analyses identified innovative target genes such as *Kif11*, *Ccna2*, and *Aurkb*. We also identified the top 10 hub genes that included *Prc1*,* Ube2c*,* Ccnb2*,* Ncapg*,* Aurkb*,* Cep55*,* Mki67*,* Dlgap5*,* Ccna2*, and *Kif11.* RT-qPCR analysis for *Tnfa*,* Il6*,* Ccl2*, and *Ccl3* further validated the RNA-seq analysis. Consistent with gene expression results, we detected significantly increased protein levels of various inflammatory mediators such as IL-6, CCL2, CXCL2, and CXCL10 in the infected lungs.

**Conclusions:**

This is the first transcriptomic analysis of the lungs of wild-type mice infected with a clinical isolate of SARS-CoV-2. Our findings provide a further understanding of the pathogenic events that occur in this mouse model of SARS-CoV-2 infection.

**Supplementary Information:**

The online version contains supplementary material available at 10.1186/s42826-025-00264-4.

## Background

Since December 2019, severe acute respiratory syndrome coronavirus 2 (SARS-CoV-2) has caused approximately 641 million infections and more than 6.6 million deaths worldwide [1]. During the initial months of the COVID-19 pandemic, SARS-CoV-2 showed minimal adaptability and phenotypic changes compared to its subsequent evolution. However, after October 2020, several variants of SARS-CoV-2 started to appear in separate regions [2]. These variants are generally characterized by unique mutations in different parts of the virus, particularly in the spike protein [3, 4]. They also exhibit changes in pathogenicity, response to the vaccines and mortality rate [5, 6]. To date, the World Health Organization has identified five SARS-CoV-2 variants as major variants of concern (VOCs) [7]. The Beta variant (B.1.351) has been the most lethal SARS-CoV-2 variant isolated to date [8, 9]. There are eight variations in the spike protein of the B.1.351 variant that can impact the affinity of the receptor-binding domain (RBD) [10, 11]. These changes enable the virus to avoid the effects of antibodies and to bind strongly to the angiotensin converting enzyme (ACE2) receptor involved in invading the host cell [10, 11]. Additionally, studies have shown less efficacy of the approved COVID-19 vaccines against this variant [12, 13].

As the ancestral SARS-CoV-2 strains did not utilize murine Ace2 as a receptor, wild-type mice were not susceptible to the SARS-CoV-2 infection. While the human ACE2-expressing mouse models serve as the primary model for studying the pathogenesis associated with SARS-CoV-2 infection, they have some disadvantages. Genetically modified mice exhibit neuroinvasive phenomena that are not present in wild-type mice [14–16]. For instance, SARS-CoV-2 infection of K18-hACE2 mice induces fatal encephalitis which is not commonly observed in humans. We and others have previously reported that the Beta variant of SARS-CoV-2 can productively infect wild-type mice and induces a strong immune response in the lungs [17, 18]. In the present study, we conducted whole transcriptomic sequencing of the lungs of wild-type C57BL/6J mice infected with the SARS-CoV-2 Beta variant. Additionally, we utilized various bioinformatics tools, such as CytoHubba, to provide a comprehensive understanding of the molecular signatures associated with SARS-CoV-2 infection. We performed RT-qPCR and multiplex immunoassay to further validate the transcriptomic analysis.

## Methods

### SARS-CoV-2 infection studies in wild-type C57BL/6J mice

C57BL/6J mice were purchased from the Jackson Laboratory. The Institutional Animal Care and Use Committee at Georgia State University authorized these studies (approved protocol number A24003). All studies involving infectious virus were performed in an approved animal biosafety level 3 facility (ABSL-3) at GSU. As described previously, C57BL/6J mice (*n* = 3–4 mice/group) were given PBS (control) or 10^4^ plaque-forming units (PFU) of SARS-CoV-2 B.1.351 that was obtained from BEI Resources (SB, BEI# NR-54008) intranasally [18]. On days 3 and 6 post-infection, isoflurane was used to render mice unconscious, and they were subsequently perfused with cold 1X PBS. The lung samples were obtained and flash-frozen in 2-methylbutane (Sigma, St. Louis, MO, USA) for further analysis. An equal number of male and female mice was used for each group.

### Extraction of RNA and RT‒qPCR

The lung tissues were homogenized in RLT buffer and total RNA was extracted using a Qiagen RNeasy Mini Kit (Qiagen, Germantown, MD, USA). The RNA quality and concentration were determined by measuring the A260/280 and A260/230 absorbance ratios using a NanoDrop spectrophotometer. Subsequently, 1,000 ng of RNA was reverse transcribed into cDNA using a Bio-Rad iScript cDNA Synthesis Kit (Catalog# 1708891). Viral load was measured with primers and probes specific for the SARS-CoV-2 N gene (Integrated DNA Technologies, Catalog# 10006713). Next, viral genome copies were determined using a standard curve and expressed per µg of total RNA. Gene expression levels were determined using the SsoAdvanced Universal SYBR Green Supermix (Bio-Rad, Catalog# 1725271). Gene expression was normalized against that of the *Gapdh* gene. The primer sequences used for RT-qPCR are listed in Table 1 [19].

**Table 1:** Primer sequences used for RT-qPCR

Gene (Accession No.)	Primer Sequence (5’-3’)
*Ccl2* (NM_011333)	Forward- TCACCTGCTGCTACTCATTCACCA Reverse- TACAGCTTCTTTGGGACACCTGCT
*Tnfa* (NM_010502)	Forward- CTCTGTGCTTTCCTGATG Reverse- CTGAGGTTATGAGTCTGAG
*Il6* (NM_000600)	Forward- CCAGGAGCCCAGCTATGAAC Reverse- CCCAGGGAGAAGGCAACTG
*Ccl3* (NM_011337)	Forward- ATTCCACGCCAATTCATC Reverse- ATTCAGTTCCAGGTCAGT

### Transcriptome profiling analysis

For this study, RNA-seq and library preparation were performed by Plasmogen. The stability of the total RNA was shown by an RNA integrity number (RIN) > 7.0 on the 2100 Bioanalyzer (Agilent Technologies, Inc., Santa Clara, CA, USA). An ultraviolet spectrophotometer (Eppendorf, Hamburg, Germany) was used to measure the amount of RNA. The TruSeq Stranded mRNA Sample Preparation Guide and TruSeq Stranded mRNA LT Sample Prep Kit were used to make a library. Lastly, the Illumina platform was used to do transcriptome analysis. The UCSC mm10 genome was used as a guide to map the cDNA snippets that were obtained from RNA sequencing [20]. A reference genome model was made with StringTie and the known genes and transcript [21]. The read count and the standardized value of fragments per kilobase of transcript per million mapped reads (FPKM) were used to determine the gene expression levels. A Limma Voom package in Galaxy Portal was used to perform the differentially expressed genes (DEGs) analysis [22, 23]. A Benjamini–Hochberg correction method with an adjusted p-value of less than 0.05 was used to find the false detection rate. Genes with an adjusted p-value below 0.05 and a log2 fold change greater than 0.75 were classified as significantly upregulated, while those with a log2 fold change less than − 0.75 were considered significantly downregulated. The “cluster profiler” and “KEGGREST” packages were also used for gene enrichment analysis, and “heatmaps,” “ggplot2,” and “ggcorrpolt” were used to visualize the results [24, 25].

### Transcriptome alignment and mapping statistics

For this study, Plasmogen constructed and sequenced 10 cDNA libraries. There were more than 40 million raw and clean reads in each library. The GC content ranged from 47 to 50 with the base percentage of the Q20 exceeding 93% and the percentage of the Q30 base surpassing 92%. In summary, the sequencing data were suitable for subsequent analysis.

### Protein-protein interaction analysis

Cytoscape 3.10 software was used to make a Protein-Protein Interaction (PPI) network, and MCODE was used to find groups [26, 27]. The hub genes from the shared DEGs were determined using the CytoHubba plugin [28].

### Cytokine and chemokine protein measurement

Infected and control lungs were homogenized using a Fisherbrand™ Bead Mill 24 Homogenizer (Fisher Scientific, Catalog# 15-340-163) and 1.4 mm ceramic beads (Fisher Scientific, Catalog# 15-340-153) for 30 s. Next, tissue homogenates were centrifuged at 10,000 RPM for 10 min. Lung homogenates were analyzed for cytokines and chemokines using the Milliplex Mouse Cytokine/Chemokine Magnetic Bead Panel (Millipore Sigma, Catalog# MCYTMAG70PMX25BK). The sample concentrations were calculated using the Belysa^®^ Immunoassay Curve Fitting Software (Millipore Sigma) [29].

### Statistically analysis of the data

GraphPad Prism version 10 was used to do the statistical tests. Statistical significance was determined as follows *p < 0.05, **p < 0.01, and ***p < 0.001.

## Results

### Comprehensive analysis of DEGs in the lungs after SARS-CoV-2 infection

**Fig. 1:**
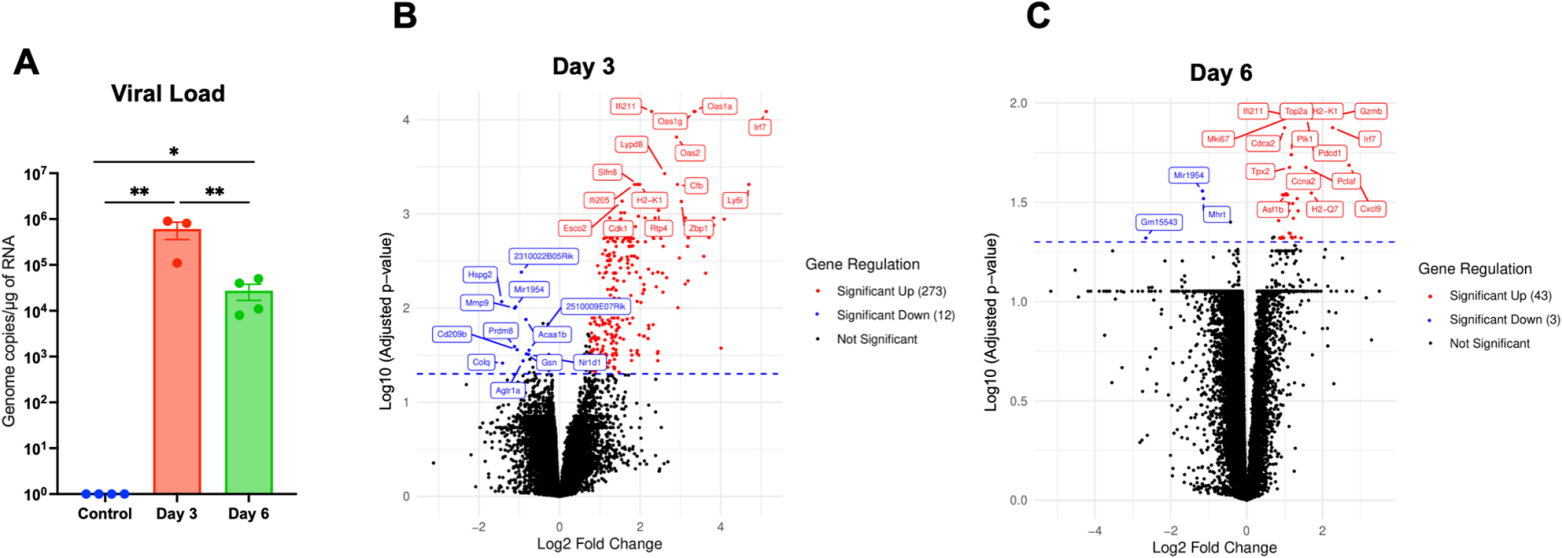
Viral load and volcano plots of DEGs in the lungs.** (A)** RT-qPCR was used to quantify SARS-CoV-2 RNA levels at days 3 and 6 post-infection. The data are expressed as genome copies/µg of RNA. Data are presented as mean ± SEM. Each data point represents an individual mouse. Statistical significance was determined with one-way ANOVA followed by a Dunnett’s multiple comparison test. *, *p* < 0.05; **, *p* < 0.001 **(B)** Volcano plot of DEGs at day 3 and **(C)** day 6 post-infection

The viral load in the infected tissues was assessed using RT-qPCR (Fig. 1A). The results demonstrated the presence of SARS-CoV-2 RNA in the lungs at days 3 and 6 post-infection. Notably, viral RNA levels were significantly lower on day 6 compared to day 3. The primary results of DEGs, as presented in Figs. 1A-B, demonstrated significant differences between the control- and virus-infected groups **(**Supplementary Tables 1–2**)**. In addition, genes with an adjusted p-value below 0.05 and a log2 fold change greater than 0.75 were classified as significantly upregulated, while those with a log2 fold change less than − 0.75 were considered significantly downregulated. Figure 1B revealed 285 DEGs at day 3, with 273 upregulated and 12 downregulated genes. At day 6, we found 46 DEGs, with 43 upregulated and 3 downregulated genes (Fig. 1C). Notably, the number of DEGs decreased significantly by day 6 post-infection. This observation is consistent with the decreased viral load in the lungs at day 6 post-infection.

**Fig. 2:**
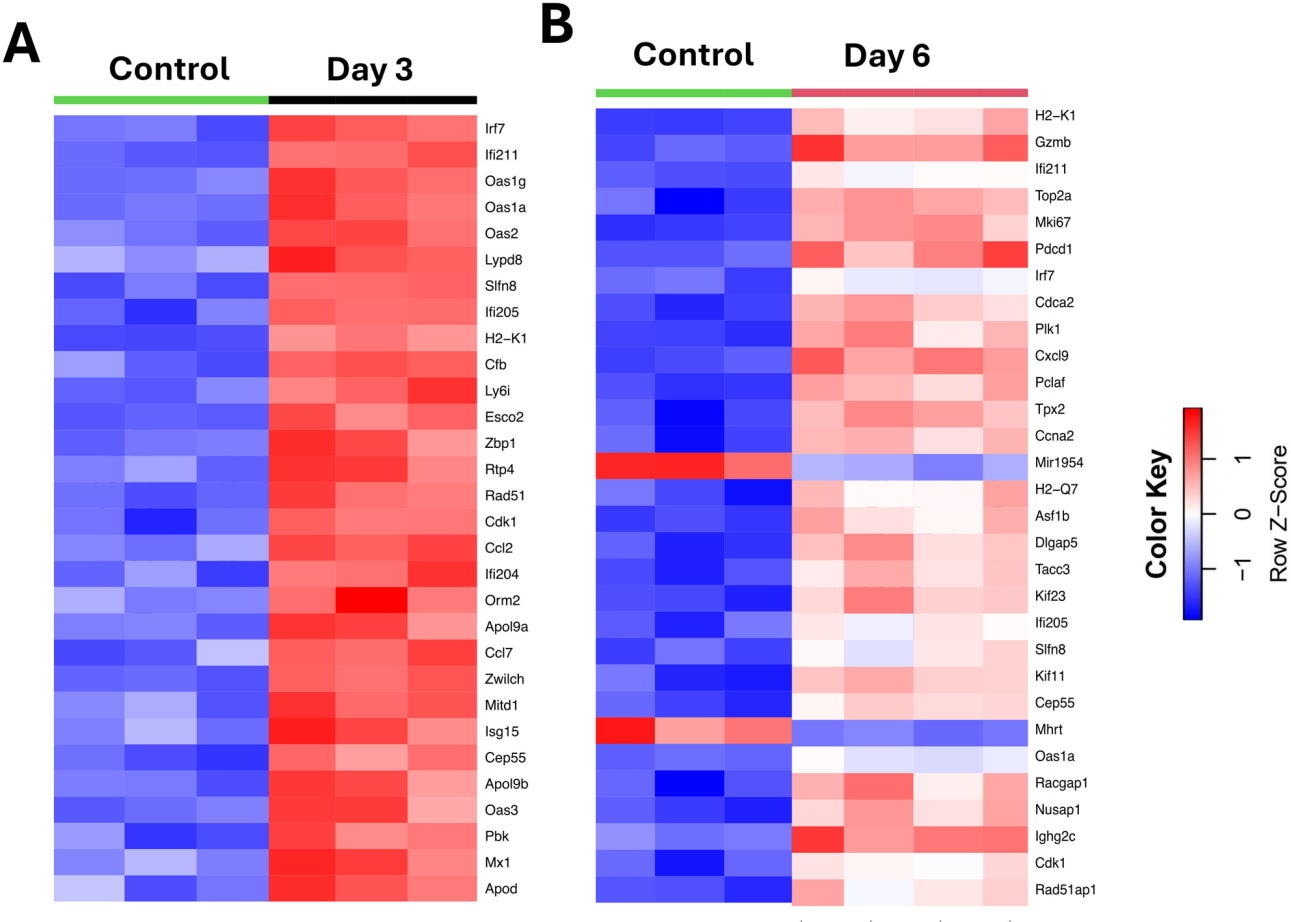
Differentially expressed genes in infected lungs. Heatmap of the top 30 differentially expressed genes with the smallest adjusted p-values (Adj. P. Val.) in SARS-CoV-2-infected mice at **(A)** day 3 vs. control and **(B)** day 6 vs. control. The color represents the level of expression based on the row Z-score: the redder the color is, the greater the gene expression, while the blueness of the color is related to lower gene expression

We next generated a heatmap of the top 30 genes based on the highest expression variance across samples from row variance of normalized counts for each time point to determine gene patterns across samples (Fig. 2A-B). On day 3 post-infection, the gene expression pattern significantly differed from that of the control group. There was a robust increase in genes associated with the interferon pathway (*Mx1*,* Irf7*,* Oas1a*,* Oas1g*,* Oas2*,* Oas3*,* Ifi204*,* Ifi205*,* and Ifi211*), the chemokine response pathway (*Ccl2 and Ccl7*) and cell cycle-related genes (*Zbp1*, *Pbk*,* Rad51*,* and Esco2*). Furthermore, on day 6 post-infection, there was a significant difference in gene expression related to cell death pathways such as *Gzmb*,* Pbcd1*,* Cdca2*,* and Top2a.* This observation is consistent with the notion that inflammatory responses are typically initiated early (day 3) as part of the host’s innate defense mechanisms. These inflammatory responses can then contribute to secondary tissue damage and immune-mediated cell death observed at later stages (day 6).

### GO and KEGG pathway enrichment analysis

**Fig. 3:**
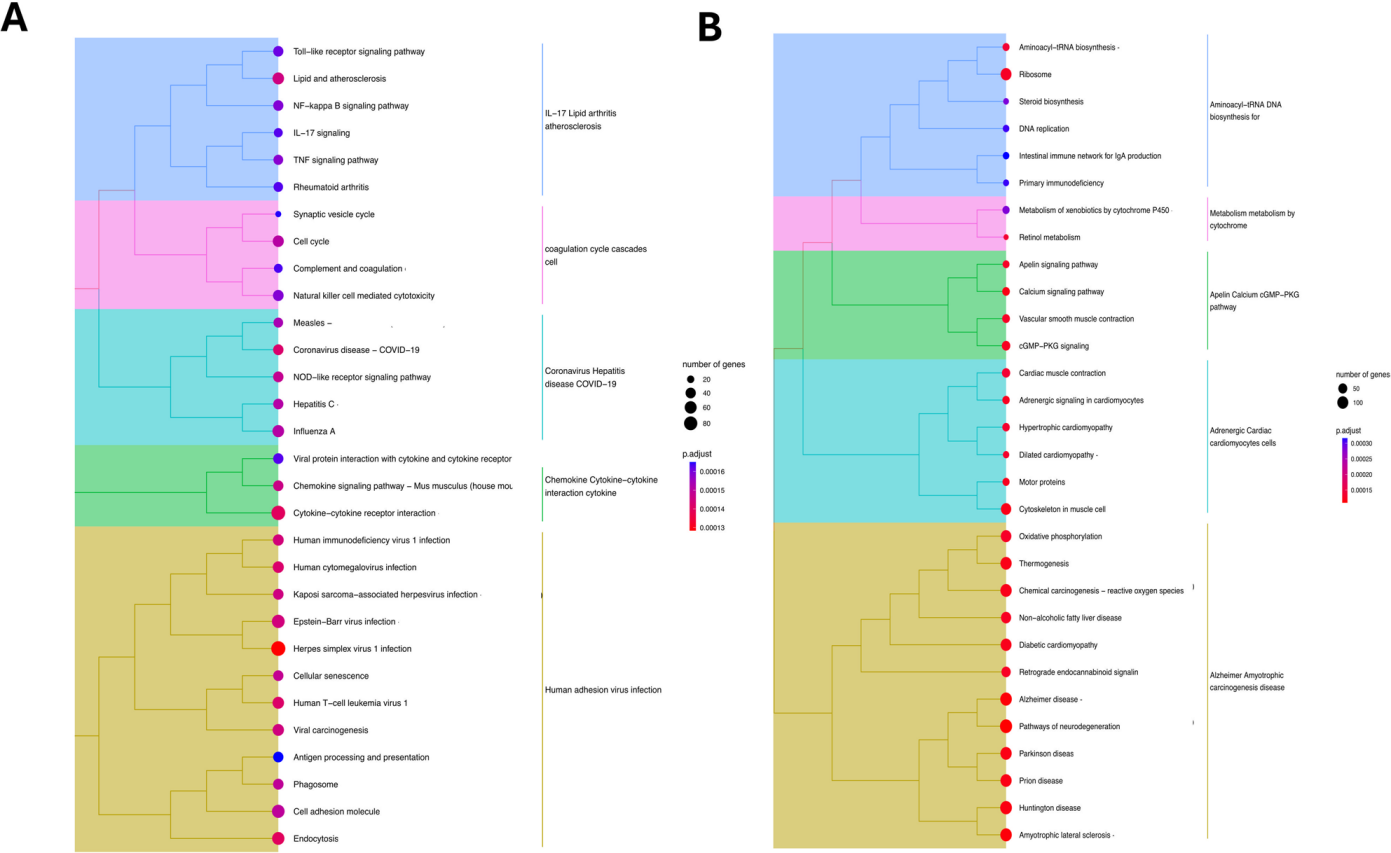
KEGG enrichment analysis. Panel **(A)** depicts the KEGG analysis of the top 30 enriched pathways identified in the lungs at day 3 post-infection, and panel **(B)** depicts the KEGG analysis of the top 30 enriched pathways identified in the lungs at day 6 post-infection

GO enrichment analysis showed that there are major changes in the genes related to structure of the extracellular matrix, myofibrils and sarcomeres after the infection. Genes linked to hydrolase and oxidoreductase activity also undergo changes **(**Supplementary Tables 3–4**)**. Previous studies have shown significant changes in the extracellular matrix, myofibril, and deposition of collagen and fibronectin in the lungs following SARS-CoV-2 infection in humans [31–33]. The canonical pathways analysis revealed the upregulation of several key pathways at day 3, such as endocytosis, cytokine and chemokine responses, toll-like receptor signaling, NOD-like receptor signaling, TNF signaling, and cellular senescence pathways (Fig. 3A). On day 6, we observed the enrichment of some notable pathways, such as calcium signaling, oxidative phosphorylation, and carcinogenesis (Fig. 3B). The TNF and NF-κB pathways have been shown to play an important role in the induction of downstream inflammatory and apoptotic responses during SARS-CoV-2 infection [34–37]. Similarly, the induction of various apoptotic genes is commonly observed in the lungs of SARS-CoV-2-infected humans and animals [38, 39].

**Fig. 4:**
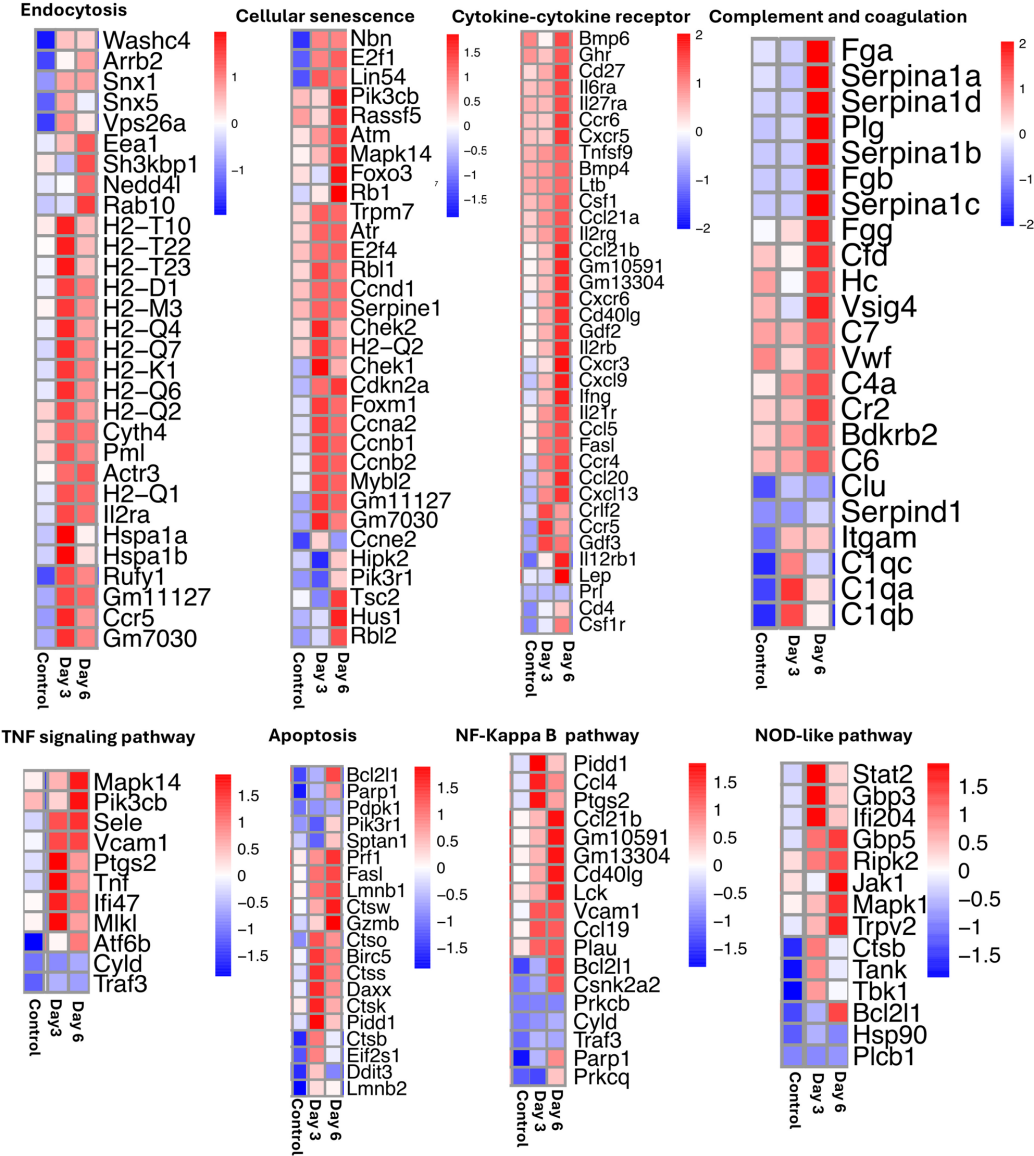
Gene Expression of Key Immune and Signaling Pathways. Heatmap of leading genes related to endocytosis, cellular senescence, cytokine receptor interaction, complement, TNF signaling, apoptosis, NF-kappa B signaling, and NOD-like receptor signaling. The color represents the level of expression based on the row Z-score. In the color scale, increased redness corresponds to higher levels of gene expression, whereas increased blueness indicates lower gene expression levels

Next, we performed targeted pathway-level analysis to identify biologically relevant genes that were altered at each time point based on functional categorization and cumulative enrichment of genes, rather than row expression variance alone. The heatmaps of genes connected to various important pathways are shown in Fig. 4. We observed a robust host response with upregulation of genes related to endocytosis, cellular senescence, cytokine-cytokine receptor (*Ccr5*, *Cxcr5*,* Gdf3*,* and crif2*), TNF and NF-κB signaling (*Tnf*,* Ifi47*, and *Mlkl*), apoptosis (*Daxx*,* FasL*, and *Gzmb*), and NOD-like receptor signaling (*Gbp5*,* Ripk2*, and *Trpv2*) at both days 3 and 6. Additionally, these results showed that genes associated with apoptosis and pyroptosis were not uniformly upregulated at a single time point, but rather showed staggered activation, with some genes upregulated at day 3 and others at day 6. Interestingly, there was more upregulation of genes associated with complement and coagulation (*Fga*,* Serpina1c*, and *C6*) at day 6 post-infection.

### Elucidating the shared and unique molecular signatures through DEG network analysis

**Fig. 5:**
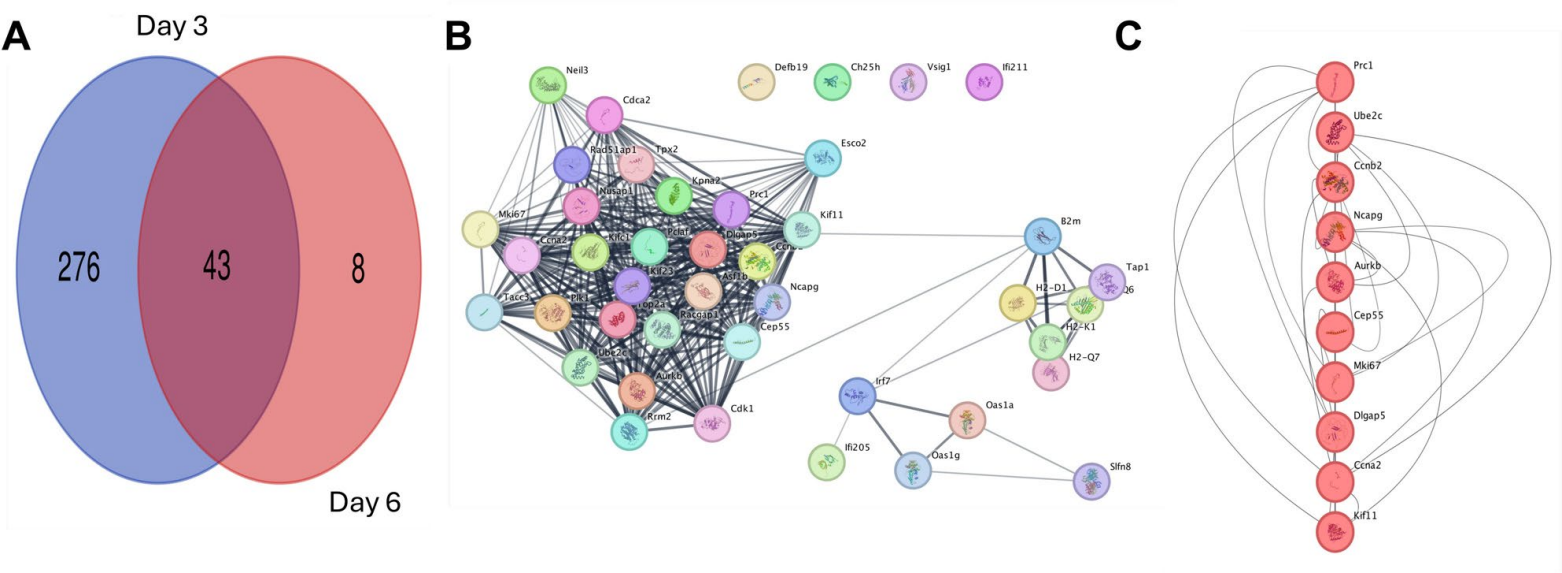
PPI Network Analysis and Hub Gene Identification.** (A)** A Venn diagram illustrating the overlap DEGs between two time points. **(B)** PPI network depicting 76 overlapping genes with three distinct clusters identified using MCODE. **(C)** The top 10 hub genes from the overlapping DEGs identified using the CytoHubba plugin

The comparative analysis of DEGs exhibited distinct patterns in gene expression, with 43 common DEGs altered on both days 3 and 6 (Fig. 5A). Protein‒protein interaction (PPI) analysis revealed three separate groups of proteins that interact with each other (Fig. 5B). These groups may represent important functional groups or pathways that are active following infection. The CytoHubba plugin’s maximal clique centrality (MCC) algorithm was used to identify the top 10 hub genes. Key genes such as *Prc1*,* Ube2c*,* Ccnb2*,* Ncapg*,* Aurkb*,* Cep55*,* Mki67*,* Dlgap5*,* Ccna2*, and *Kif11* are highlighted in Fig. 5C.

### Experimental validation of RNA-seq data

**Fig. 6:**
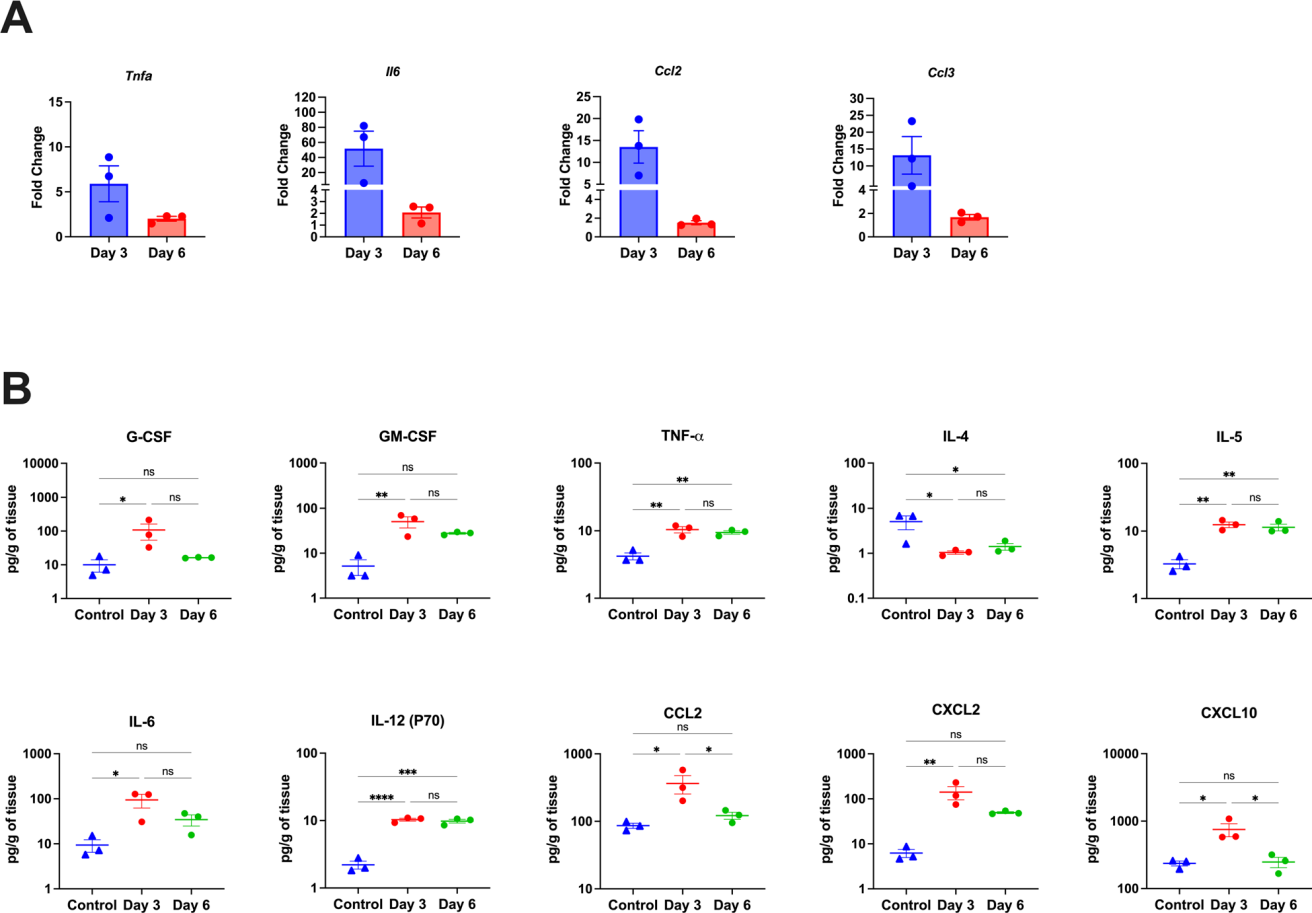
Experimental validation of RNA-Seq.** (A)** RNA levels of *Tnfα*, *Il6*, *Ccl2*, and *Ccl3* were measured by RT-qPCR. Expression levels are represented as fold change relative to control group after normalization with the housekeeping gene. **(B)** Cytokine and chemokine protein levels in control and infected lungs at 3- and 6-days post-infection. Data are presented as mean ± SEM. Each point represents an individual mouse. Statistical significance was determined by a one-way ANOVA or Kruskal-Wallis test, followed by Dunn’s or Tukey’s multiple comparisons test for unpaired data sets. (*p < 0.05; ** *p* < 0.01; *** *p* < 0.001, **** *p* < 0.0001)

Next, RT-qPCR was used to measure the RNA levels of the key genes identified in the RNA-seq analysis. The gene expression results aligned with the RNA-seq data, demonstrating high levels of *Tnfa*, *Il6*, *Ccl2*, and *Ccl3* in the infected group compared to the controls. Furthermore, for all genes, the expression levels on day 3 were higher than on day 6 (Fig. 6A). Next, we performed a multiplex immunoassay to measure the protein levels of several cytokines and chemokines in the infected lungs. Consistent with gene expression results, we detected significantly increased protein levels of IL-6, CCL2, CXCL2, and CXCL10 in the lungs at day 3 post-infection. Additionally, we detected significantly increased levels of G-CSF and GM-CSF in infected lungs at day 3 post-infection. Notably, at day 6 post-infection, we detected slightly higher levels of IL-6, CCL2, and CXCL2 compared to control, but the difference was not statistically significant. Levels of TNF-α, IL-5, and IL-12 (P70) were significantly elevated in the infected lungs at both time points. On the other hand, levels of IL-4 were significantly decreased in the infected lungs (Fig. 6B).

## Discussion

Ancestral SARS-CoV-2 B.1 does not infect wild-type mice because of the differences in the sequence of murine Ace2 and human ACE2 [30]. To address this, several transgenic mice expressing human ACE2 have been developed to study SARS-CoV-2 pathogenesis. However, because of the abnormal expression of ACE2, these mice experience fatal neuroinflammation, a characteristic absent in humans infected by SARS-CoV-2 [31, 32]. Our study provides a unique perspective by investigating the pathogenesis of the SARS-CoV-2 Beta variant (B.1.351) in wild-type C57BL/6J mice, which do not overexpress ACE2, making them a more physiologically relevant model for studying lung-specific immune responses.

We and others have previously shown that the SARS-CoV-2 Beta variant (B.1.351) can productively infect wild-type mice and induce inflammation in the lungs [18]. In the present study, by employing RNA-seq analysis, we explored the pathogenesis of B.1.351 infection in the lungs. Differential gene expression analysis revealed that SARS-CoV-2 infection modulates the expression of several key genes in the lungs. Our findings are consistent with previous studies using ACE2-transgenic models, such as K18-hACE2 mice, which exhibited robust immune responses to SARS-CoV-2 variants including, Delta, Beta, and Alpha, characterized by the upregulation of key immune genes (Cxcl10, Zbp1, Ifit3, Isg15, Rsad2, and Irf7) [33]. In addition, pathway analysis showed these genes are associated with various pathways known to be crucial for COVID-19 pathogenesis, including endocytosis, TNF signaling, NF-kappa B signaling, apoptosis, cytokine response, and NOD-like receptor signaling pathways [18]. For example, endocytosis plays an important role in the pathogenesis of many viruses by facilitating their entry into the cytoplasm [34]. On the other hand, TNF plays a very important role in shaping anti-viral immunity against SARS-CoV-2 [35]. Additionally, NF-κB pathway activation has been shown to modulate inflammatory response during COVID-19 [36, 37]. In response to a viral infection, apoptosis acts as a defensive mechanism by eliminating the virus-infected cell, thereby preventing the virus from spreading to other cells [38]. Our previous study demonstrated that infection with the Beta variant resulted in the most extensive gene dysregulation among the variants tested, characterized by strong activation of inflammatory pathways associated with the acute-phase response [33]. Although the severity of infection in wild-type mice was lower than that observed in ACE2-transgenic models, Beta variant infection still triggered a pronounced hyperinflammatory response, marked by elevated expression of proinflammatory cytokines and chemokines.

Protein–protein interaction (PPI) network analyses identified several hub genes, including *Prc1*, *Ube2c*, *Ccnb2*, *Ncapg*, *Aurkb*, *Cep55*, *Mki67*, *Dlgap5*, *Ccna2*, and *Kif11*, that may contribute to SARS-CoV-2 pathogenesis [39, 40]. These genes are primarily involved in regulating the cell cycle, mitotic spindle formation, chromosomal segregation, and DNA replication. Recent studies using various biological data have shown that *Prc1* and *Ccnb2* are dysregulated in both COVID-19 patients and lung fibrosis disorders like silicosis [41, 42]. Similarly, large-scale transcriptomic analyses have consistently highlighted *Ube2c*, *Kif11*, and *Aurkb* as key genes modulated in severe COVID-19 patients, especially in immune tissues and reproductive systems [43]. The dysregulation of *Mki67*, a well-known marker for cell growth, suggests that SARS-CoV-2 infection can impact the cell cycle. Also, the previous reports showed that SARS-CoV-2 infection of human lung epithelial cells activates strong interferon and cytokine responses, with widespread changes in proteins related to immune activation and cell regulation [44, 45]. Together, these findings underscore a shared pathogenic landscape between COVID-19 and other proliferative or fibrotic lung diseases. They also provide a foundation for identifying novel biomarkers and therapeutic targets aimed at modulating virus-induced immune dysregulation and tissue damage.

## Conclusions

In summary, our study provides a transcriptomic analysis of the lungs of wild-type C57BL/6J mice infected with a non-mouse-adapted clinical isolate of SARS-CoV-2 (B.1.351). The differential gene expression patterns showed a robust immune response against SARS-CoV-2 with the upregulation of several innate and inflammatory pathways. The use of a wild-type mouse model, rather than an ACE2-transgenic model, provides a unique perspective on SARS-CoV-2 pathogenesis that may closely reflect the host immune response in the absence of transgenic ACE2 expression. These results highlight the importance of using physiologically relevant models to study viral pathogenesis and host immune responses.

## Supplementary Information

Below is the link to the electronic supplementary material.

Supplementary Material 1

Supplementary Material 2

Supplementary Material 3

Supplementary Material 4

## Data Availability

The original contributions presented in the study are included in the article/ Supplementary Material; further inquiries can be directed to the corresponding author.

## Abbreviations

ACE2: Angiotensin-converting enzyme 2
Ccl2: C-C motif chemokine ligand 2
Ccl3: C-C motif chemokine ligand 3
DEGs: Differentially expressed genes
FPKM: Fragments per kilobase of transcript per million mapped reads
Il6: Interleukin 6
MCC: Maximal Clique Centrality
PPI: Protein-Protein Interaction
RBD: Receptor-binding domain
SARS-CoV-2: Severe acute respiratory syndrome coronavirus 2
Tnfa: Tumor necrosis factor alpha
VOCs: Variants of concern
